# Working Memory Training Coupled With Transcranial Direct Current Stimulation in Older Adults: A Randomized Controlled Experiment

**DOI:** 10.3389/fnagi.2022.827188

**Published:** 2022-04-12

**Authors:** Ana C. Teixeira-Santos, Célia S. Moreira, Diana R. Pereira, Diego Pinal, Felipe Fregni, Jorge Leite, Sandra Carvalho, Adriana Sampaio

**Affiliations:** ^1^Psychological Neuroscience Laboratory, Psychology Research Centre, School of Psychology, University of Minho, Braga, Portugal; ^2^Department of Social Sciences, Institute for Research on Socio-Economic Inequality, University of Luxembourg, Esch-Belval, Luxembourg; ^3^Department of Mathematics, Centre for Mathematics of the University of Porto, Faculty of Sciences, University of Porto, Porto, Portugal; ^4^Spaulding Neuromodulation Center, Department of Physical Medicine and Rehabilitation, Harvard Medical School, Boston, MA, United States; ^5^Portucalense Institute for Human Development, Universidade Portucalense, Porto, Portugal; ^6^Translational Neuropsychology Lab, Department of Education and Psychology and William James Center for Research (WJCR), University of Aveiro, Campus Universitário de Santiago, Aveiro, Portugal

**Keywords:** tDCS, cognitive training, working memory, neuroplasticity, older adults, reasoning, transfer effects

## Abstract

**Background:**

Transcranial direct current stimulation (tDCS) has been employed to boost working memory training (WMT) effects. Nevertheless, there is limited evidence on the efficacy of this combination in older adults. The present study is aimed to assess the delayed transfer effects of tDCS coupled with WMT in older adults in a 15-day follow-up. We explored if general cognitive ability, age, and educational level predicted the effects.

**Methods:**

In this single-center, double-blind randomized sham-controlled experiment, 54 older adults were randomized into three groups: anodal-tDCS (atDCS)+WMT, sham-tDCS (stDCS)+WMT, and double-sham. Five sessions of tDCS (2 mA) were applied over the left dorsolateral prefrontal cortex (DLPFC). Far transfer was measured by Raven’s Advanced Progressive Matrices (RAPM), while the near transfer effects were assessed through Digit Span. A frequentist linear mixed model (LMM) was complemented by a Bayesian approach in data analysis.

**Results:**

Working memory training improved dual *n*-back performance in both groups submitted to this intervention but only the group that received atDCS+WMT displayed a significant improvement from pretest to follow-up in transfer measures of reasoning (RAPM) and short-term memory (forward Digit Span). Near transfer improvements predicted gains in far transfer, demonstrating that the far transfer is due to an improvement in the trained construct of working memory. Age, formal education, and vocabulary score seem to predict the gains in reasoning. However, Bayesian results do not provide substantial evidence to support this claim.

**Conclusion:**

This study will help to consolidate the incipient but auspicious field of cognitive training coupled with tDCS in healthy older adults. Our findings demonstrated that atDCS may potentialize WMT by promoting transfer effects in short-term memory and reasoning in older adults, which are observed especially at follow-up.

## Introduction

Working memory training (WMT) has been proposed as a prominent intervention in older adults, which may benefit not only WM but other cognitive processes related to it (e.g., reasoning), which is called far transfer effects (Teixeira-Santos et al., 2019). Specifically, older people seem to have benefits from the training of updating tasks, in which participants hold specific content in memory, continually updating the information to be remembered and dropping information that is no longer needed (Pergher et al., 2018). Updating is reduced in old age and it mediates age-related differences in reasoning (Chen and Li, 2007; Jaeggi et al., 2008).

The results of different studies of WMT are controversial with gains mostly being observed in near transfer (i.e., in other WM tasks) but not so commonly in far transfer tasks (Melby-Lervåg and Hulme, 2016; Teixeira-Santos et al., 2019). Transcranial direct current stimulation (tDCS) is a well-tolerated non-invasive brain stimulation technique, in which a weak direct current is applied through the cerebral cortex *via* electrodes that are placed upon the scalp. tDCS modulates the resting membrane potential, introducing variations in the response threshold of the neuron and modifying neuronal synaptic efficiency. It is involved with mechanisms of long-term potentiation and long-term depression, resulting in effects that outlast the stimulation period (Nitsche and Paulus, 2000; Nitsche et al., 2003). Most of the studies that used tDCS as an add-on intervention to WMT have been conducted with young adults and have reported that anodal-tDCS (atDCS) over the dorsolateral prefrontal cortex (DLPFC) may improve WM (Fregni et al., 2005; Zaehle et al., 2011). Nonetheless, other studies failed to find such results following a single-tDCS session (Horvath et al., 2015). Although most of the studies so far were performed in a single-tDCS session, the literature suggests a beneficial effect of repeated sessions (Martin et al., 2013).

Even though few studies have combined WMT with tDCS in older adults, the results of some studies have pointed to improvements on the trained task and transfer measures, with the effects normally delayed, verified specially at follow-up sessions (Park et al., 2013; Jones et al., 2015; Stephens and Berryhill, 2016). However, Nilsson et al. (2017) failed to find transfer effects in a 20-session tDCS when coupled with an executive functioning training. The same research group failed to find a superiority effect of 1 and 2 mA atDCS (vs. sham-tDCS – stDCS) in an *n*-back task that was performed in a single session (Nilsson et al., 2015). As tDCS effects in older adults may be mainly observed in an extended follow-up yet not immediately after training (Jones et al., 2015), this may be the explanation for these discrepant findings. Even so, more studies are necessary to clarify it.

Furthermore, individual differences seem to interact with WMT and tDCS effects (Berryhill and Jones, 2012; Gözenman and Berryhill, 2016; Borella et al., 2017; Ruf et al., 2017; Di Rosa et al., 2019; Ke et al., 2019). In special, two factors have been shown to be predictive of cognitive training gains: baseline cognitive ability and age (Zinke et al., 2014; Guye et al., 2017; Shaw and Hosseini, 2021). Gender and educational level are also proved as predictors (Roheger et al., 2020, 2021). The influence of those factors may be based on two hypotheses: the compensation and the magnification effects. Following Shaw and Hosseini (2021), the compensation hypothesis states that individuals with high baseline performance, less age, and high educational level will benefit less from training because they are already nearest to the optimal level of functioning and, therefore, there is less room for improvement in this ability. On the other hand, the magnification hypothesis postulates the opposite, i.e., high-performing, high-educational level, and young participants will take more advantage of training, since they already performed well. Accordingly, these participants may be even more efficient on the task after training, as they have more cognitive resources that will boost the training. The same authors also emphasize that even though the magnification hypothesis has been corroborated by WMT literature, many studies also presented evidence in favor of the compensation hypothesis. Specifically, in studies that associate cognitive training with tDCS in older adults, there is more evidence in favor of the compensation hypothesis, in which training gains were mainly observed in low-cognitive-performance participants (Perceval et al., 2020; Krebs et al., 2021). Evidence in favor of the compensation hypothesis in tDCS studies has also been verified in young adults (Habich et al., 2017).

Therefore, in this study, we assessed the effects of a 5-day tDCS coupled with dual *n*-back training, an update task simultaneously tapping verbal and visuospatial modalities of WM, immediately after training and in a 15-day follow-up in older adults. Our hypothesis is that far transfer would be verified only in the atDCS+WMT group and at follow-up. An additional analysis explored whether baseline performance, general cognitive ability, age, and educational level predicted the effects.

## Materials and Methods

A Consolidated Standards of Reporting Trials (CONSORT) diagram is presented in Supplementary Figure 1. The CONSORT is a set of initiatives to improve the quality of clinical trial reports (Moher et al., 2001). In this context, participants and assessors were blinded to stimulation and task conditions. To assure blindness, stimulation and assessments were performed by different researchers. The researcher who applied the tDCS and set the computer task was not blind. The randomization list was generated on a website^1^ in blocks of six participants with a ratio of 2:2:2. The allocation list was masked from all investigators. The condition of each participant was described in different excel sheets. The researcher responsible for data collection only had access to the allocation of the next participant in the first session. The study was performed in accordance with the Declaration of Helsinki and approval was obtained from the ethics subcommittee for life and health sciences of the University of Minho (SECVS 012/2016). Participation was voluntary. Participants gave informed consent before their inclusion in the study.

### Participants

The eligibility criteria were as follows: (1) be right-handed, (2) with normal or corrected-to-normal visual and auditory acuity, (3) with no history of neurological/psychiatric disorders, substance abuse, or recent use of psychotropic medication, and (4) no contraindication for tDCS. Participants were recruited in senior daycare centers and in recreation clubs in the North of Portugal. See Supplementary Table 1 for group comparison at baseline. In the screening session, participants completed a socioeconomic and clinical questionnaire, the Jaeger Card, an auditory discrimination letters task, the Geriatric Anxiety Inventory (GAI) (Ribeiro et al., 2011), the Geriatric Depression Scale (GDS) (Pocinho et al., 2009), and the Montreal Cognitive Assessment (MoCA) (Freitas et al., 2011). We screened 108 participants, from which 54 were excluded. The reasons for exclusion were the presence of neurological or psychiatric disorder history, hearing problems, being below the threshold score in MoCA and GDS, metal in the head, and non-availability to enroll in the study. All participants scored above the 2 standard deviations (SDs) cutoff for their age and education group in the Portuguese adaptation of the MoCA and scored below 9 in GDS.

For sample size calculation, we assumed a medium effect and estimated the sample size using the R package WebPower (Zhang et al., 2018). Since we were planning to analyze data through mixed-effects models, having the interaction “group × moment” as fixed effect and participants as random effects (factorial mixed design), we selected a repeated measure ANOVA with within-between interaction effect power analysis. Indeed, it accounts for both within- and between-subject effects and, from the statistical point of view, it is based on the same logic and the same parameters (Perugini et al., 2018). Then, the sample size calculation was proceeded after considering three groups, three measurements, 80% of power, 5% of type I error probability, and Cohen’s *d* = 0.5 [or Cohen’s *f* = 0.204, for this particular case (Cohen, 1988, p. 278)]. Considering an attrition rate of 6.5% (Basak and O’Connell, 2016), 54 participants were enrolled (18 in each arm). Therefore, 54 participants (68.20 ± 5.92 years old, 41 women) were randomized to one of three groups: (1) atDCS+WMT (i.e., anodal-tDCS + dual *n*-back training); (2) stDCS+WMT (i.e., sham-tDCS + dual *n*-back training); and (3) double-sham (i.e., sham-tDCS + placebo task: a visuoperceptual task). All participants randomized completed the study.

### Procedures

Participants underwent 11 sessions that were held individually. The first session was the screening for inclusion criteria and three sessions (the second, the ninth, and the eleventh) were assessment sessions. In two sessions (the third and the tenth), participants underwent an electroencephalogram (EEG) recording, the results of which will be discussed in another manuscript. Finally, from session four to session eight, participants underwent the stimulation sessions. During the pretest session, participants performed the Vocabulary and Digit Span subtests of the Wechsler Adult Intelligence Scale – Third Edition (WAIS-III) (Wechsler, 2008), the Raven’s Advanced Progressive Matrices (RAPM) (Raven et al., 1998), and a dual *n*-back task. In the posttest, immediately after training and at the 15 days follow-up, participants performed the Digit Span test, the RAPM, and the dual *n*-back. In addition, the blinding was assessed in the follow-up session.

During training, participants underwent five sessions of 20 min each. The trained task (dual *n*-back or the placebo task) and the tDCS condition (sham or anodal) were selected according to the randomization sequence, which is described in the participants’ subsection. Participants answered a Visual Analog Scale (VAS) to assess possible tDCS side effects, before and after each day of the intervention. The VAS items assessed levels of discomfort, fatigue, anxiety, pain, itching, humor, tingling, headache, and sleepiness. To test participants’ blinding, participants were asked to guess in which condition they were allocated regarding task and tDCS conditions.

**FIGURE 1:**
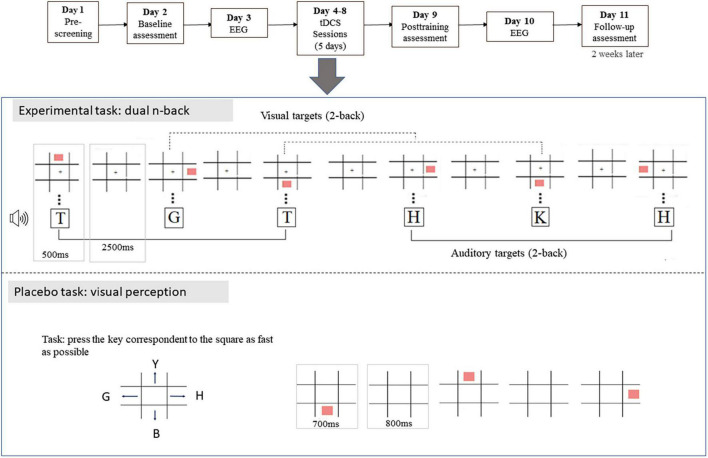
Schematic representation of the sessions. The experimental task was illustrated for a 2-back condition. All participants underwent 11 sessions, as represented in the top of this figure. Participants from atDCS+WMT and stDCS+WMT groups performed the dual *n*-back task while participants from the double-sham group performed the visual perception task.

Figure 1 depicts the experimental and control tasks. The atDCS+WMT and the stDCS+WMT groups performed the dual *n*-back task during training. The dual *n*-back task was displayed using the Presentation software package (Neurobehavioral Systems, Albany, CA, United States). During this task, participants were simultaneously shown visuospatial and auditory-verbal stimuli. The visuospatial stimulus was a square presented in one of eight possible locations in a 3 × 3 grid with a fixation cross on the central square. The auditory-verbal stimulus was one of nine possible consonants (T, G, X, H, R, S, L, K, and J), delivered binaurally through Sony MDR-NC6 noise-canceling headphones in a random order. Stimuli were presented for 500 ms, with 2,500 ms of interstimulus interval. In each trial, participants decided whether the stimulus displayed was the same presented *n* trials before. Participants were instructed to press the “spacebar” every time either a visuospatial or an auditory-verbal target was a match. At the end of each block, a feedback with the participant’s hits was shown. The task consisted of 12 blocks with 25 trials each. In each block, there were two auditory-verbal and two visuospatial targets, and one stimulus that was a target in both modalities. The *n* level started with *n* = 1 and increased by 1 if the participants achieved 100% of hits in three consecutive blocks. During training, the *n* level started at the maximum level achieved by the participant in the previous day. If the number of hits in the last three blocks of the previous session was inferior to 60%, then the *n* level was decreased by 1.

A simple visual perceptual task was used for sham training in order to control confounding variables that resulted from the intervention setting and to allow the blinding of participants (see Figure 1). The double-sham group performed this placebo task, which was presented in SuperLab software (The Experimental Laboratory Software, version 5.0.3; Cedrus Corporation, San Pedro, CA, United States). In this task, a 3 × 3 grid was presented with a fixation cross in the center. Squares were presented in one of four possible locations in the grid. Participants had to press the arrow key corresponding to the position where the square showed up. There were 203 trials in total. Stimuli duration was 700 ms, with 800 ms of interstimulus interval.

#### Transcranial Direct Current Stimulation Parameters

In the anodal condition, tDCS was applied for 20 min with an intensity of 2 mA, using two 5 × 7 cm^2^ rectangular saline-soaked electrode sponges with anode positioned over the left DLPFC (F3) and the cathode over the contralateral supraorbital area (Fp2). The F3 is the area commonly used to place the anode electrode (Fregni et al., 2005; Zaehle et al., 2011; Martin et al., 2013; Nilsson et al., 2015, 2017) due to its broad connections to cortical and subcortical structures and its relationship with WM (D’Esposito et al., 2000; Levy and Goldman-Rakic, 2000). Besides that, DLPFC has an active role in the active manipulation of information during WM processing (Smith and Jonides, 1999; Collette and Van Der Linden, 2002). The current was applied with a 15 s fade-in at the beginning and a 15-s fade-out at the end of the stimulation. Participants started the cognitive task after 3 min of tDCS. For the sham condition, the electrode setup was identical to the anodal condition. However, the stimulation was discontinued after 45 s of administration (15 s of fade-in/stimulation/fade-out). Stimulation techniques were applied using a Magstim Eldith DC Stimulator Plus (NeuroConn, Ilmenau, Germany, DE).

### Outcome Measures and Predictor Variables

The far transfer outcome, assessing reasoning, was the RAPM_sets 1 and 2. The reasoning was used due to its strong relationship with WM and its common use in the field of WMT (Conway et al., 2003; Chen and Li, 2007; Oberauer et al., 2008). The RAPM is composed of 48 figures within a 3 × 3 matrix of geometrical shapes, in which one of the shapes was missing. By choosing from eight options, participants were asked to complete the missing part of the figure. Two parallel forms were set by separating odd and even trials. The versions were randomized and counterbalanced between sessions in a way that in pretest and follow-up participants used one form, while in the posttest, they performed the other version. One point was given for each correct response, and the outcome was the sum of correct responses (maximum score of 6 points for set 1 and 18 for set 2). No time restriction was imposed.

In the domain of near transfer, we measured working memory using Digit Span. During this task, participants listened to a sequence of digits and were instructed to recall them in the forward and backward orders. The first two trials consisted of two digits and the length of the sequence was increased by 1 every two trials. When the participant had two consecutive errors at the same level, the task was discontinued. The outcome was the total number of sequences correctly recalled separately in the forward and backward modalities (maximum score of 16 for forward order and 14 for backward order).

We used the following variables as predictors of WMT gains: the change in the scores of near-transfer measures (i.e., ‘posttest minus baseline’ and ‘follow-up minus baseline’ of forward and backward Digit Span measures’), age (in years), years of formal education, and general cognitive ability (operationalized by RAPM_set 2 and Vocabulary scores).

### Data Analysis

Statistical analyses were conducted using RStudio, version 3.5.2 and the packages: lme4 (Bates et al., 2015); lmerTest (Kuznetsova et al., 2017); glmmTMB (Brooks et al., 2017); brms (Bürkner, 2017); ordinal (Christensen, 2019); effects (Fox and Weisberg, 2018); and lsmeans (Lenth, 2016). We ran mixed-effects models in the analyses due to their flexibility and efficiency in analyzing repeated measures, accounting for pretest differences in the outcomes (Winter, 2013). The level of significance was set at 0.05. We confirmed our results with Bayesian analysis. We rejected the null hypothesis for the values of *p* close to 0.05 when the Bayesian analyses pointed out evidence in favor of the alternative hypothesis (Dienes, 2011), namely, when the posterior probability was equal or bigger than 0.95. In other words, to be considered significant, results should have a *p* < 0.05 or a *PP* ≥ 0.95. No adjustment for multiple comparisons was done since we have used linear mixed model (LMM) instead of classical procedures, such as ANOVA. Models yield more efficient estimates, shifting estimates toward each other and making comparisons more conservative (Gelman et al., 2012).

The primary analysis was performed with the RAPM from the follow-up session, since we were mainly interested in verifying if atDCS+WMT yielded larger far transfer effects when compared to stDCS+WMT and double-sham groups at follow-up. The secondary analysis considered the performance changes in dual *n*-back and the scores on Digit Span at posttest, Digit Span at follow-up, and RAPM at posttest.

Effect sizes were calculated using the package “metafor” (Viechtbauer, 2010), taking into consideration the pretest performance. They were calculated using Hedges’ *g* (Hedges, 1989) presented by Morris (2008), in formula 5, with a bias correction presented in formula 22. Based on Cohen (1988), *g* = 0.2 was considered small, *g* = 0.5 was considered medium, and *g* = 0.8 was considered large.

All outcomes were dichotomous (gender), ordered (the maximum level achieved during training), or discrete variables (task scores). For dichotomous outcomes, we performed logistic models (binomial distribution). For ordered categorical data, we conducted ordered logit models. For the discrete outcomes, we performed Conway–Maxwell–Poisson models. The ordered logit models were obtained with the R package “ordinal” (Christensen, 2019) and all the others were obtained with the R package “glmmTMB” (Brooks et al., 2017). Bayesian models used the default for Markov chains, with 2,000 iterations per chain (such as warmup).

## Results

Only small side effects (i.e., skin redness, small burning sensation, and itching) were reported following stimulation. There was no group difference in VAS assessed in all sessions to verify tDCS side effects (see Supplementary Table 2). Regarding blinding assessments, no differences were found between the three groups. Most participants believed they were performing the WMT. This was the case for 78% of participants in the atDCS+WMT group, 72% of participants of stDCS+WMT, and 83% of participants from the double-sham group. Most participants also believed they were receiving atDCS. This was the case for 89% of participants from the atDCS+WMT group, 94% of participants from the stDCS+WMT group, and 100% of participants from a double-sham group.

Mixed models were analyzed to verify the relationship between each outcome (i.e., RAPM_sets 1 and 2 and Digit Span forward and backward order) in the three assessment moments (pretest, posttest, and follow-up), considering the stimulation condition (atDCS+WMT, stDCS+WMT, double-placebo, and dual *n*-back task). We entered into the model the interaction group × moment as fixed effect and participants as a random effect. The performance of the dual *n*-back task (probability of maximum level achieved across the 5 days of training) of the atDCS+WMT and stDCS+WMT groups was assessed with a similar approach; however, the “moment” variable was considered a continuous variable (from 1 to 5).

**FIGURE 2:**
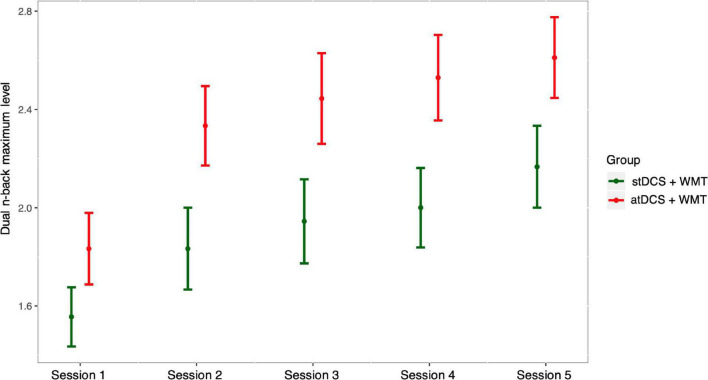
Dual *n*-back maximum level. The horizontal axis represents the five training sessions while the vertical axis represents the raw data with the maximum level participants achieved in the dual *n*-back task in that session.

The analysis of the gains observed during training in the dual *n*-back task showed that training had a positive effect on the performance of the participants from both groups (atDCS+WMT and stDCS+WMT). Between two consecutive sessions, the odds of upgrading a level was increased about three times along the sessions [exp (1.09) = 2.97, *p* < 0.001], i.e., rating in higher levels was more likely along with the sessions. There was a group effect at the baseline showing that the odds of achieving a given level versus its lower levels were about 18 times higher for the stDCS+WMT group [exp (2.89) = 17.99, *p* = 0.045] than for the atDCS+WMT group. There was no interaction effect of group × moment [exp (0.10) = 1.11, *p* = 0.75]. Figure 2 and Supplementary Figure 2 show the raw and fitted data of dual *n-*back maximum level, respectively. In short, trained groups were improved during the sessions with no significant difference between them, although the WMT+stDCS group started the training to achieve a higher level than the atDCS+WMT.

**FIGURE 3:**
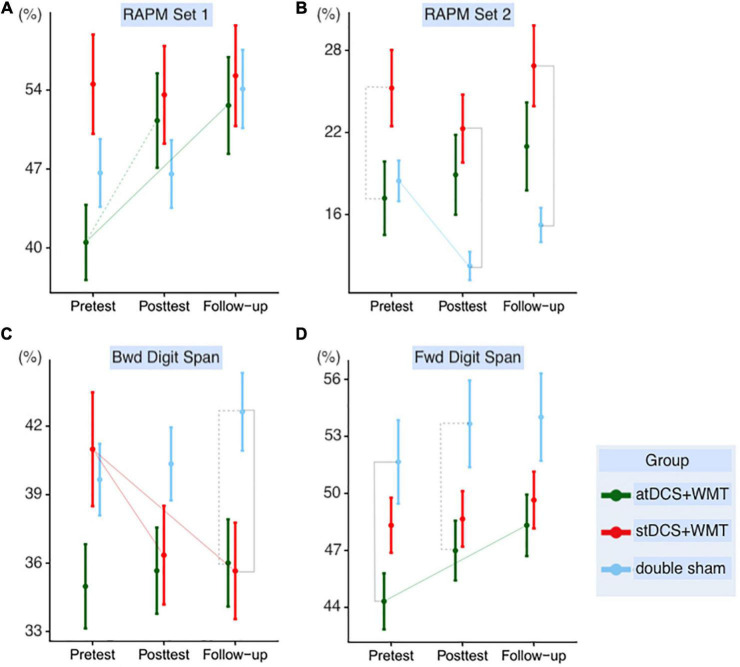
Fitted data representation of group × session interaction for each outcome. The vertical axis represents the percentage of hits regarding the total possible hits. Segments represent the 95% CIs. Solid lines represent statistically significant differences (*p* < 0.05), while dashed lines represent marginally significant results (*p* < 0.1) supported by Bayesian posterior probabilities (PP ≥ 0.95). RAPM_set 1 **(A)**; RAPM_set 2 **(B)**; backward Digit Span **(C)**; forward Digit Span **(D)**. atDCS, active tDCS; stDCS, sham tDCS; RAPM, Raven Advanced Progressive Matrices.

The main interest of this study was related to the far transfer effects of the stimulation. Therefore, as previously mentioned, we ran LMMs to the different outcomes (RAPM_sets 1 and 2, forward and backward Digit Span) at posttest and follow-up to verify if there were differences between the groups over time. In what follows, results are described for each outcome in the following sequence: first, between-moments significant differences were listed for each group (see Figure 3 and Table 1); second, for each moment, between-groups differences were highlighted (see Table 2); and third, we presented the Hedges’ *g* for each analysis (see Supplementary Table 3). In Supplementary Material, we present Supplementary Table 4 for descriptive statistics of each outcome by group and assessment session and Supplementary Table 5 for the correlations between pretest and posttest and between pretest and follow-up.

**TABLE 1:** Generalized mixed-effect models result for each moment per group.

			Frequentist analysis			Bayesian analysis				
Outcome	Group	Moment comparison	Estimate	SE	*p*-Value	Estimate	EE	CI	BF	*PP*
RAPM_set 1	atDCS+WMT	posttest – Pretest	0.23	0.13	**0.076^∧^**	0.20	0.12	]0.01, ∞[	22.67	0.96*
		Follow-up – Pretest	0.25	0.13	0.047*	0.22	0.12	]0.03, ∞[	32.61	0.97*
		Follow-up – posttest	0.02	0.12	0.834	0.02	0.11	]0.03, ∞[	1.32	0.57
	stDCS+WMT	posttest – Pretest	–0.02	0.12	0.884	–0.02	0.11	]0.03, ∞[	1.31	0.57
		Follow-up – Pretest	0.01	0.11	0.909	0.02	0.11	]0.03, ∞[	1.27	0.56
		Follow-up – posttest	0.03	0.12	0.795	0.03	0.11	]0.03, ∞[	1.64	0.62
	Double-sham	posttest – Pretest	0.00	0.13	0.987	0.00	0.11	]0.03, ∞[	1.00	0.50
		Follow-up – Pretest	0.14	0.12	0.238	0.13	0.11	]0.03, ∞[	7.49	0.88
		Follow-up – posttest	0.14	0.12	0.232	0.13	0.11	]0.03, ∞[	8.78	0.90
RAPM_set 2	atDCS+WMT	posttest – Pretest	0.09	0.16	0.597	0.08	0.17	]−0.02, ∞[	2.22	0.69
		Follow-up – Pretest	0.18	0.16	0.254	0.17	0.16	]−0.09, ∞[	5.62	0.85
		Follow-up – posttest	0.09	0.15	0.539	0.09	0.16	]0.17, ∞[	2.57	0.72
	stDCS+WMT	posttest – Pretest	–0.12	0.14	0.418	–0.11	0.15	]−∞, 0.13[	3.48	0.78
		Follow-up – Pretest	0.06	0.14	0.670	0.06	0.14	]−0.17, ∞[	1.98	0.66
		Follow-up – posttest	0.17	0.14	0.217	0.17	0.15	]−0.06, ∞[	7.40	0.88
	Double-placebo	posttest – Pretest	–0.36	0.18	0.041*	–0.36	0.18	]−∞, −0.07[	49.00	0.98*
		Follow-up – Pretest	–0.17	0.17	0.305	–0.17	0.18	]−∞, 0.12[	4.76	0.83
		Follow-up – posttest	0.19	0.18	0.300	0.19	0.19	]−0.12, ∞[	5.58	0.85
Backward DS	atDCS+WMT	posttest – Pretest	0.02	0.07	0.734	0.02	0.07	]−0.09, ∞[	1.73	0.63
		Follow-up – Pretest	0.03	0.07	0.612	0.03	0.06	]−0.07, ∞[	2.24	0.69
		Follow-up – posttest	0.01	0.07	0.870	0.01	0.06	]−0.09, ∞[	1.30	0.57
	stDCS+WMT	posttest – Pretest	–0.14	0.07	0.029*	–0.13	0.06	]−∞, −0.03[	51.63	0.98*
		Follow-up – Pretest	–0.17	0.07	0.012*	–0.15	0.07	]−∞, −0.05[	116.65	0.99*
		Follow-up – posttest	–0.02	0.07	0.736	–0.02	0.07	]−∞, 0.09[	1.62	0.62
	Double-sham	posttest – Pretest	0.02	0.06	0.750	0.02	0.06	]−0.08, ∞[	1.59	0.61
		Follow-up – Pretest	0.04	0.06	0.177	0.08	0.06	]−0.02, ∞[	10.14	0.91
		Follow-up – posttest	0.06	0.06	0.301	0.06	0.06	]−0.04, ∞[	5.16	0.84
Forward DS	atDCS+WMT	posttest – Pretest	0.06	0.04	0.163	0.06	0.04	]−0.02, ∞[	9.67	0.91
		Follow-up – Pretest	0.09	0.04	0.038*	0.08	0.04	]0.01, ∞[	42.48	0.98*
		Follow-up – posttest	0.03	0.04	0.497	0.03	0.04	]−0.04, ∞[	2.86	0.74
	stDCS+WMT	posttest – Pretest	0.01	0.04	0.865	0.01	0.04	]−0.06, ∞[	1.26	0.56
		Follow-up – Pretest	0.03	0.04	0.503	0.03	0.04	]−0.04, ∞[	2.75	0.73
		Follow-up – posttest	0.03	0.04	0.618	0.02	0.04	]−0.05, ∞[	2.12	0.68
	Double-sham	posttest – Pretest	0.04	0.04	0.329	0.04	0.04	]−0.03, ∞[	4.85	0.83
		Follow-up – Pretest	0.05	0.04	0.252	0.04	0.04	]−0.02, ∞[	6.37	0.86
		Follow-up – posttest	0.01	0.04	0.865	0.01	0.04	]−0.06, ∞[	1.24	0.55

^∧^p < 0.1; *p < 0.05 or PP ≥ 0.95.

*Values of p (frequentist analysis) and posterior probability (Bayesian analysis) are indicated. Significant values in bold. CI – 95% credible interval. BF, Bayes Factor (evidence ratio); DS, Digit Span; EE, estimate error; PP, posterior probability; RAPM, Raven’s Advanced Progressive Matrices; SE, standard error.*

**TABLE 2:** Generalized mixed-effects models results for between-group analysis per moment.

			Frequentist analyses			Bayesian analysis				
Outcome	Moment	Group comparison	Estimate	SE	*p*-Value	Estimate	EE	CI	BF	*PP*
RAPM_set 1	Pretest	stDCS+WMT – atDCS+WMT	0.28	0.18	0.118	0.24	0.17	]−0.04, ∞[	11.86	0.92
		Double-sham – atDCS+WMT	0.15	0.18	0.401	0.13	0.17	]−0.16, ∞[	3.41	0.77
		Double-sham – stDCS+WMT	–0.13	0.17	0.468	–0.11	0.16	]−∞, 0.15[	3.03	0.75
	posttest	stDCS+WMT – atDCS+WMT	0.03	0.17	0.843	0.02	0.17	]−0.25, ∞[	1.22	0.55
		Double-sham – atDCS+WMT	–0.08	0.17	0.660	–0.07	0.17	]−∞, 0.20[	2.01	0.67
		Double-sham – stDCS+WMT	–0.11	0.17	0.524	–0.09	0.16	]−∞, 0.18[	2.48	0.71
	Follow-up	stDCS+WMT – atDCS+WMT	0.04	0.17	0.818	0.04	0.16	]−0.23, ∞[	1.41	0.59
		Double-sham – atDCS+WMT	0.04	0.17	0.802	0.04	0.16	]−0.22, ∞[	1.55	0.61
		Double-sham – stDCS+WMT	0.00	0.17	0.983	0.01	0.16	]−0.26, ∞[	1.12	0.53
RAPM_set 2	Pretest	stDCS+WMT – atDCS+WMT	0.41	0.23	**0.076^∧^**	0.39	0.24	]0.00, ∞[	19.94	0.95*
		Double-sham – atDCS+WMT	0.16	0.24	0.500	0.16	0.25	]−0.26, ∞[	2.71	0.73
		Double-sham – stDCS+WMT	–0.25	0.23	0.272	–0.24	0.24	]−∞, 0.16[	5.46	0.85
	posttest	stDCS+WMT – atDCS+WMT	0.21	0.23	0.365	0.20	0.24	]−0.20, ∞[	4.01	0.80
		Double-sham – atDCS+WMT	–0.29	0.25	0.243	–0.28	0.26	]−∞, 0.14[	6.72	0.87
		Double-sham – stDCS+WMT	–0.50	0.24	0.040*	–0.48	0.25	]−∞, −0.08[	38.60	0.97*
	Follow-up	stDCS+WMT – atDCS+WMT	0.29	0.22	0.199	0.28	0.24	]−0.11, ∞[	7.95	0.89
		Double-sham – atDCS+WMT	–0.19	0.24	0.418	–0.19	0.25	]−∞, 0.22[	3.28	0.77
		Double-sham – stDCS+WMT	–0.48	0.23	0.038*	–0.47	0.24	]−∞, −0.08[	39.40	0.98*
Backward DS	Pretest	stDCS+WMT – atDCS+WMT	0.18	0.11	0.100	0.17	0.11	]−0.01, ∞[	15.95	0.94
		Double-sham – atDCS+WMT	0.16	0.11	0.143	0.15	0.11	]−0.02, ∞[	11.99	0.92
		Double-sham – stDCS+WMT	–0.02	0.11	0.858	–0.02	0.11	]−∞, 0.15[	1.28	0.56
	posttest	stDCS+WMT – atDCS+WMT	0.02	0.11	0.877	0.02	0.11	]−0.16, ∞[	1.23	0.55
		Double-sham – atDCS+WMT	0.16	0.11	0.150	0.15	0.11	]−0.03, ∞[	11.62	0.92
		Double-sham – stDCS+WMT	0.14	0.11	0.198	0.13	0.11	]−0.05, ∞[	8.73	0.90
	Follow-up	stDCS+WMT – atDCS+WMT	–0.02	0.11	0.881	–0.02	0.11	]−∞, 0.16[	1.26	0.56
		Double-sham – atDCS+WMT	0.21	0.11	**0.054^∧^**	0.20	0.10	]−0.03, ∞[	32.90	0.97*
		Double-sham – stDCS+WMT	0.23	0.11	0.038*	0.22	0.11	]−0.04, ∞[	42.01	0.98*
Forward DS	Pretest	stDCS+WMT – atDCS+WMT	0.09	0.07	0.193	0.09	0.07	]−0.03, ∞[	8.15	0.89
		Double-sham – atDCS+WMT	0.15	0.07	0.030*	0.14	0.07	]0.03, ∞[	53.79	0.98*
		Double-sham – stDCS+WMT	0.06	0.07	0.383	0.06	0.07	]−0.06, ∞[	3.63	0.78
	posttest	stDCS+WMT – atDCS+WMT	0.04	0.07	0.586	0.03	0.07	]−0.08, ∞[	2.28	0.69
		Double-sham – atDCS+WMT	0.13	0.07	**0.059^∧^**	0.12	0.07	]0.01, ∞[	25.32	0.96*
		Double-sham – atDCS+WMT	0.09	0.07	0.179	0.09	0.07	]−0.03, ∞[	8.37	0.89
	Follow-up	stDCS+WMT – atDCS+WMT	0.03	0.07	0.668	0.03	0.07	]−0.09, ∞[	1.99	0.66
		Double-sham – atDCS+WMT	0.11	0.07	0.116	0.10	0.07	]−0.01, ∞[	12.89	0.93
		Double-sham – stDCS+WMT	0.08	0.07	0.254	0.07	0.07	]−0.04, ∞[	6.01	0.86

^∧^p < 0.1, *p < 0.05 or PP ≥ 0.95.

*Values of p (frequentist analysis) and posterior probability (Bayesian analysis) are indicated. Significant values in bold. CI – 95% credible interval. BF, Bayes Factor (evidence ratio); DS, Digit Span; EE, estimate error; PP, posterior probability; RAPM, Raven’s Advanced Progressive Matrices; SE, standard error.*

In the analysis of RAPM_set 1, a marginally significant difference between the pretest and posttest (*p* = 0.076, *PP* = 0.96) and a significant difference between the pretest and the follow-up session (*p* = 0.047, *PP* = 0.97) were found only for the atDCS+WMT group, with no significant group differences in each moment (*p* = 0.12, *PP* = 0.92). The Hedges’ *g* for group differences between atDCS+WMT and stDCS+WMT was medium (*g* = 0.6) at posttest and follow-up. In the case of the comparison between atDCS+WMT and double-sham group, the effect size was medium at posttest (*g* = 0.5) but small at follow-up (*g* = 0.3). Therefore, atDCS+WMT outperformed both groups at posttest and follow-up. Regarding stDCS+WMT vs. the double-sham, the former displayed lower transfer effects in both posttest (*g* = −0.03) and follow-up (*g* = −0.26).

In the analysis of the RAPM_set 2, the double-sham group performed lower at posttest as compared to pretest (*p* = 0.041, *PP* = 0.98). Regarding the differences between groups in each moment, the stDCS+WMT outperformed the double-sham group at posttest (*p* = 0.040, *PP* = 0.97) and follow-up (*p* = 0.038, *PP* = 0.98). atDCS+WMT started with a marginally significant lower performance when compared to the stDCS+WMT at pretest (*p* = 0.076, *PP* = 0.95). However, this difference was no longer significant at posttest/follow-up (*p* = 0.199, *PP* = 0.97), indicating an improvement in the atDCS+WMT group. Hedges’ *g* for group differences between atDCS+WMT and stDCS+WMT was small at posttest (*g* = 0.3) and follow-up (*g* = 0.2). For the comparison between atDCS+WMT and double-sham, the effect size was large at posttest (*g* = 0.9) and medium at follow-up (*g* = 0.6), showing that transfer effects were superior in atDCS+WMT when compared to the two other groups at posttest and follow-up. When compared stDCS+WMT and the double-sham, the Hedges’ *g* was medium at both moments (*g*_posttest_ = 0.6; *g*_follow–up_ = 0.5).

Considering the near transfer analysis, for the backward Digit Span, LMMs showed a difference between pretest and posttest (*p* = 0.029, *PP* = 0.98) and between pretest and follow-up (*p* = 0.012, *PP* = 0.99) for the stDCS+WMT group, in which this group had their performance decreased over the sessions. Regarding the differences between groups, the performance of the double-sham was marginally superior to that of atDCS+WMT (*p* = 0.054, *PP* = 0.97) and significantly superior to that of double-sham at follow-up (*p* = 0.038, *PP* = 0.98). atDCS+WMT demonstrated a superior small Hedges’ *g* when compared to the stDCS+WMT at posttest (*g* = 0.4) and a medium effect size at follow-up (*g* = 0.5). When compared with the double-sham, the effect was closer to zero at posttest (*g* = −0.01) and small at follow-up (*g* = −0.2). In the case of stDCS+WMT vs. the double-sham, the effect sizes were negative (*g*_posttest_ = −0.4; *g*_follow–up_ = −0.7).

In the forward Digit Span analysis, LMMs showed a difference between the pretest and the follow-up only for the atDCS+WMT group (*p* = 0.038, *PP* = 0.98). With respect to the group differences, atDCS+WMT performed lower than the double-sham at the pretest (*p* = 0.030, *PP* = 0.98) and posttest (*p* = 0.59, *PP* = 0.96) but this difference was no longer significant at follow-up (*p* = 0.116, *PP* = 0.93). atDCS+WMT had a superior small Hedges’ *g* as compared to the stDCS+WMT at posttest (*g* = 0.3) and follow-up (*g* = 0.3). When compared to the double-sham, the difference was closer to zero at posttest (*g* = 0.1) and small at follow-up (*g* = 0.3). When we compare stDCS+WMT with the double-sham, the effect sizes were close to zero (*g*_posttest_ = −0.12; *g*_follow–up_ = −0.05).

We performed a further analysis to verify whether gains on near transfer measures (backward and forward Digit Span) predicted gains on reasoning. This would demonstrate that the far transfer is due to an improvement in the trained construct of working memory. As such, we ran LMMs with the RAPM_set 1 scores of the atDCS+WMT group, having the gains for near-transfer measures (i.e., forward and backward Digit Span) as fixed effect and participants as a random effect. Gains were calculated as the difference between posttest and pretest scores, also with the difference between follow-up and the pretest scores. In this analysis, a transformation of the variables was made, i.e., after computing the difference between posttest (or follow-up) and pretest scores, a constant was added to each variable. As a result, we observed that gains in backward Digit Span predicted gains on reasoning (estimate = 0.10, *p* = 0.02). The analysis of forward Digit Span was not significant (*p* = 0.207) (see Supplementary Table 6).

**FIGURE 4:**
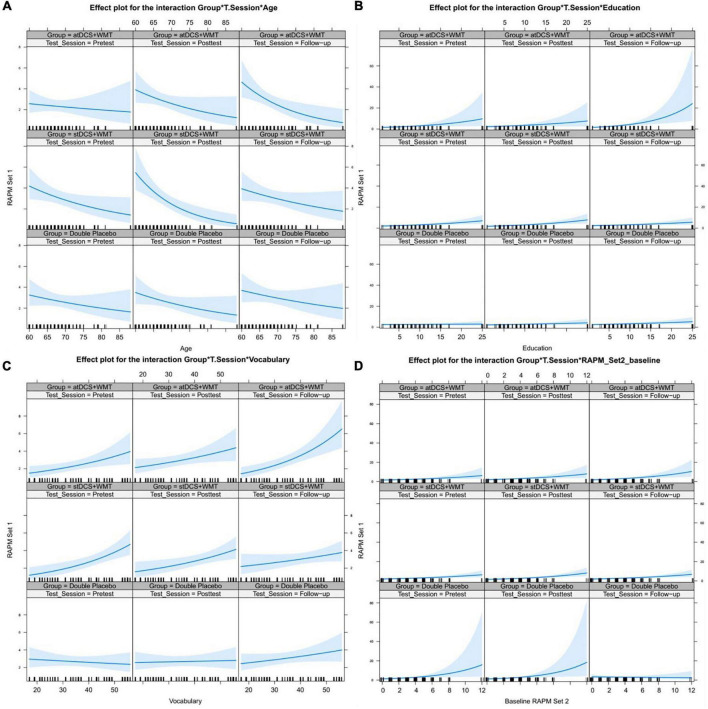
Fitted values (Group × Testing Session × Predictor) for each predictor in RAPM_set 1 scores. Predictors: age **(A)**; education **(B)**; vocabulary **(C)**; baseline RAPM_set 2 **(D)**. The shaded area is a pointwise 95% confidence band for the fitted values, based on standard errors and computed from the covariance matrix of the fitted regression coefficients using Conway-Maxwell-Poisson models.

Finally, we run an analysis to verify if individual differences would influence the WMT transfer effects on the RAPM_set 1. For such, we added the age of the predictors, educational level, general cognitive ability (operationalized by RAPM_set 2), and Vocabulary scores at baseline as fixed effect in the three-way interaction: RAPM_set 1 ∼ group × moment × predictor. As random effects, we had intercepts for participants. We performed one model for each variable. The results of these analyses are presented in Figure 4.

In Figure 4A, showing the reasoning scores as a function of age, the slope of the line is increased from pretest to follow-up (*p* = 0.045, *PP* = 0.90) in the case of the atDCS+WMT group, showing that younger participants benefited more from training than the older ones. For the stDCS+WMT group, the slope became less inclined from the posttest to follow-up (*p* = 0.022, *PP* = 0.94), i.e., young-older participants from the WMT+stDCS group had a decrease in their difference from old-older participants from posttest to follow-up, suggesting that the benefits of training were not sustained at follow-up. No significant change was observed in the double-placebo group. Figure 4B, depicting reasoning scores as a function of years of education, reveals a marginally significant increase in the slope from posttest to follow-up (*p* = 0.066) in the atDCS+WMT group, meaning that participants with more years of education improved more in reasoning score than those with low educational background. Figure 4C, which presents reasoning as a function of Vocabulary scores at baseline, demonstrates an increase of slope from posttest to follow-up in the atDCS+WMT group (*p* = 0.036, *PP* = 0.89), meaning that participants with higher Vocabulary scores improved more than those participants with lower Vocabulary scores. Conversely, for the stDCS+WMT group, the slope was decreased in the follow-up session (*p* = 0.043, *PP* = 0.88). Figure 4D, which shows reasoning scores as a function of RAPM_set 2 scores at pretest, shows no significant change in the slopes. Even though the frequentist analysis suggested the influence of age and vocabulary performance at pretest in the effects, the confidence intervals (CIs) include zero (see Supplementary Table 7). Additionally, the Bayesian analysis did not provide sufficient evidence to support these results (see Supplementary Table 6).

## Discussion

In this study, we assessed the transfer effects of atDCS coupled with WMT when compared to stDCS plus WMT or a double-sham condition in healthy older adults, immediately after training and in a 15 days follow-up. Individual characteristics were also explored as predictors of transfer effects. Regarding feasibility, we had no dropouts in this study, which may indicate that the protocol was well tolerated and suitable for our sample, validating the use of WMT with tDCS in the older population.

During training, both groups submitted to WMT (atDCS+WMT and stDCS+WMT) improved in the trained task (dual *n*-back) throughout session, having no significant difference between them, which was expected and in line with previous literature (Stephens and Berryhill, 2016). However, considering transfer effects and as pointed out in Table 1 and Figure 3, our data suggested that the atDCS+WMT was the only group that displayed a significant improvement from pretest to follow-up in transfer measures of reasoning (RAPM) and short-term memory (forward Digit Span). The transfer effects are consistent with Ruf et al. (2017) who observed near transfer effects after 3 days of DLPFC atDCS coupled with an *n*-back task. However, other tDCS studies failed to find such transfer effects (Lawlor-Savage and Goghari, 2016; Nilsson et al., 2017). These differences among studies may be due to variations in experimental protocols. To illustrate it, Nilsson et al. (2017) had a longer protocol with 20 days of intervention, whereas for another study, participants trained for 5 weeks, 5 days per week (Lawlor-Savage and Goghari, 2016). In fact, a previous meta-analysis had shown that short-period training may be more effective than long-period training (Teixeira-Santos et al., 2019). Moreover, Pergher et al. (2018) demonstrated that 5 days of WMT were sufficient to improve *n*-back performance in older adults with no difference observed between 5 or 10 days of training.

When we designed this study, we had as the main hypothesis that far transfer would be verified only in the atDCS+WMT group and at follow-up. As WMT alone does not seem to produce far transfer effects (Teixeira-Santos et al., 2019), we anticipated that tDCS could boost training, promoting the far transfer and, so, the gains in reasoning would be seen only in atDCS+WMT. Additionally, this effect would be verified at the follow-up session since previous studies found delayed effects in older adults (Park et al., 2013; Jones et al., 2015; Stephens and Berryhill, 2016). In fact, the transfer effects were stronger at follow-up than immediately after the intervention. This phenomenon is known as the “sleeper effect,” i.e., improvements in some cognitive domains take long time to manifest in older adults, likely related to the deterioration in the brain microstructures (Jaeggi et al., 2014; Borella et al., 2019). We had a follow-up period of 15 days due to feasibility reasons regarding the duration of the project. However, a more prominent effect might be verified if we had a longer follow-up period, which would be interesting to be pursued in future studies.

The stDCS+WMT outperformed the double-sham in RAPM_set 2, which is in line with previous WMT experiments (Borella et al., 2017). Unexpectedly, in backward Digit Span, the stDCS+WMT had a drop in the performance at posttest and follow-up. Probably, if we had used a WM task more similar to the trained one (i.e., an updating task), we would have found a more positive effect as transfer effects appear to be bigger when there is a greater overlap between the trained and transfer task (Byrne et al., 2020). The content between trained and transfer tasks was also different (letters for training, numbers for transfer), which increased the lack of overlap between tasks, not counting that the assessment of transfer was in a paper-and-pencil while the training was computerized (Byrne et al., 2019).

The fact that near transfer effects observed in the atDCS+WMT were restricted to forward Digit Span, not achieving improvement in the backward order may be associated with a limit in the plasticity of working memory related to the limit of short-term memory, the 7 ± 2 rule (Miller, 1956; Shaw and Hosseini, 2021). It may also be attributed to the stimulated area (F3). Forward Digit Span processing depends upon a rehearsal mechanism of working memory (phonological loop), which might benefit from stimulation at left sites, since previous pieces of evidence have been highlighting the importance of laterality-dependence of stimulation by proposing right DLPFC tDCS in the case of spatial tasks and left tDCS in the case of verbal stimuli (von Bastian and Oberauer, 2013). On the other hand, backward Digit Span demands a mental transformation that arguably left stimulation may be not able to reach. Since visual working memory decline is more accentuated in older adults, right stimulation might be more advantageous in this group (Cansino et al., 2013). Additionally, prior literature showed improvements in working memory after 1 mA atDCS (Hoy et al., 2013; Zokaei et al., 2015; Ruf et al., 2017) and greater effects in studies delivering the stimulation before the execution of the task (Cansino et al., 2013). Therefore, future studies could address the optimization of the stimulation parameters.

Interesting enough, gains in working memory performance predicted gains in reasoning, confirming the rationale that far transfer is dependent on near transfer gains (Melby-Lervåg et al., 2016). However, this relationship was restricted to backward Digit Span. This was not surprising as backward Digit Span involves additional processing demands in comparison with the forward modality, which only requires simple retention of information (Zokaei et al., 2015).

Finally, the frequentist analysis suggested that age, formal education, and vocabulary score at pretest modulated transfer effects. Strictly speaking, younger participants from the atDCS+WMT group improved more in RAPM scores than older participants throughout the testing sessions while the gains of training were lost in young-old adults from the stDCS+WMT group. This supports the potential of the tDCS to boost the maintenance of the effects. In the same direction, participants with more years of formal education from the atDCS+WMT group benefited more from training. The results having vocabulary as a predictor were less direct: whereas the atDCS+WMT participants with higher scores in the vocabulary showed more improvements in reasoning, the stDCS+WMT participants with low pretest vocabulary were the ones demonstrating more improvements. This may happen because high-vocabulary participants have small room for improvement in WMT, but when tDCS enters the equation, the WMT effects are boosted and these participants improve even more in their general cognitive abilities. Those participants might activate the neural resources more efficiently when tDCS reinforces this mechanism. In sum, those analyses are in favor of the magnification hypothesis, also known as the “*Mathew effect*,” imported from the Matthew 13:12 Biblical statement: “Whoever has will be given more, and they will have an abundance” (von Bastian and Oberauer, 2013). In other words, as young-old well-educated and high-performance participants already perform better in fluid abilities than old-old low-educated low-performance participants, this discrepancy tends to increase with the stimulation. It is important to highlight that education is not only a proxy of socioeconomic status but also of cognitive reserve (Rouillard et al., 2017) as does the Vocabulary score (Lojo-Seoane et al., 2014). Therefore, age or maybe socioeconomic and cognitive reserve may influence the stimulation outcomes in older adults. Additionally, given the complexity of the trained task, low-performance participants might have more difficulty to perform the dual *n*-back task while the high-performance participants were more motivated to do that since they could perform it better. These results of individual differences in gains are important to the implementation of more tailored interventions. However, Bayesian analyses do not provide enough evidence to confirm that individual differences predict transfer effects. Given the lack of robustness of our findings and the fact that our results point in a direction contrary to other studies associating cognitive training and tDCS in older adults (Perceval et al., 2020; Krebs et al., 2021), further studies are still necessary to verify the predictive effects of education level, age, and vocabulary.

Overall, this study stands out because it had three conditions that allowed us to separate the effects of tDCS from WMT. Instead of using a passive control group, we had an active control one. This allowed us to control the effects that resulted from social contact and participants’ expectations. The main limitation of this work was a between-group difference at baseline even though the participants were randomly allocated to the groups, which was considered by the LMM approach. Furthermore, bigger samples seem to be necessary to have satisfactory statistical power, especially considering that most studies on WMT are underpowered (Guye et al., 2017). Therefore, this study needs to be replicated in experiments having larger sample sizes.

## Conclusion

Working memory training improved WM performance in a dual *n*-back task in older adults. However, transfer effects were observed only when the WMT was coupled with atDCS and they were mainly observed at follow-up. Specifically, atDCS+WMT yielded improvement in short-term memory and reasoning, showing evidence in favor of their combined use. Future studies could directly address real-life outcomes in order to improve the ecological validity of this intervention.

## Data Availability Statement

The raw data supporting the conclusions of this article will be made available by the authors, without undue reservation.

## Ethics Statement

The studies involving human participants were reviewed and approved by Ethics Subcommittee for Life and Health Sciences of University of Minho (SECVS 012/2016). The patients/participants provided their written informed consent to participate in this study.

## Author Contributions

ACT-S and AS concepted and designed the study and worked on the acquisition of subjects. ACT-S was responsible for study execution, acquiring and coding the data, preparation of tables and figures, statistical analysis, interpreting the data, and preparing the manuscript. CSM performed the statistical analysis and preparation of the tables and figures. AS and SC contributed to the supervision of the study. FF, SC, and JL provided training in the tDCS technique. DRP helped in data collection. All authors critically revised the paper for important intellectual content.

## Conflict of Interest

The authors declare that the research was conducted in the absence of any commercial or financial relationships that could be construed as a potential conflict of interest.

## Publisher’s Note

All claims expressed in this article are solely those of the authors and do not necessarily represent those of their affiliated organizations, or those of the publisher, the editors and the reviewers. Any product that may be evaluated in this article, or claim that may be made by its manufacturer, is not guaranteed or endorsed by the publisher.

## Footnotes

1
http://www.randomization.com
