# Supplementary material for: Working Memory Training Coupled With Transcranial Direct Current Stimulation in Older Adults: A Randomized Controlled Experiment
Source: Front Aging Neurosci. 2022 Apr 12;14:827188. doi: 10.3389/fnagi.2022.827188 (PMC9039392; doi:10.3389/fnagi.2022.827188)
Supplement: Supplementary file 1 [file Data_Sheet_1.pdf]

# Working Memory Training Coupled with tDCS in Older Adults: a randomized, controlled experiment

## Appendix A. Supplementary data

Supplementary Table S1.
*Characterization of the sample.*

| Measure | atDCS +WMT | stDCS+ WMT | Double-sham | *p*-value | ER | PPS |
|---|---|---|---|---|---|---|
| Age | 67.61 (5.11) | 68.67 (6.98) | 68.33 (5.82) | G2-G1: .583 | 2.41 | .71 |
| | | | | G3-G1: .707 | 1.61 | .62 |
| | | | | G3-G2: .863 | 1.36 | .58 |
| Gender (female/male) | 14/4 | 13/5 | 14/4 | G2-G1: .701 | 1.93 | .66 |
| | | | | G3-G1: 1.00 | 1.00 | .50 |
| | | | | G3-G2: .701 | 1.98 | .66 |
| Education (in years) | 6.39 (3.26) | 8.89 (5.74) | 7 (5.95) | G2-G1: .101 | 16.86 | .94 |
| | | | | G3-G1: .657 | 1.93 | .66 |
| | | | | G3-G2: .231 | 7.02 | .88 |
| MoCA | 22.5 (2.94) | 23 (3.66) | 22.56 (1.92) | G2-G1: .753 | 1.57 | .61 |
| | | | | G3-G1: .972 | 1.02 | .51 |
| | | | | G3-G2: .780 | 1.53 | .60 |
| GAI | 3.72 (4.53) | 3.11 (3.68) | 3.67 (3.22) | G2-G1: .654 | 2.02 | .67 |
| | | | | G3-G1: .970 | 1.05 | .51 |
| | | | | G3-G2: .682 | 1.87 | .65 |
| GDS | 4.72 (3.21) | 3.56 (3.11) | 3.06 (3.13) | G2-G1: .326 | 4.87 | .83 |
| | | | | G3-G1: .138 | 14.75 | .94 |
| | | | | G3-G2: .613 | 2.30 | .70 |
| Vocabulary | 33.5 (12.30) | 41.94 (12.26) | 34.78 (11.72) | G2-G1: **.035*** | 40.24 | **.98**† |
| | | | | G3-G1: .737 | 1.66 | .62 |
| | | | | G3-G2: .076^ | 23.1 | **.96**† |

*Notes.* Mean values with standard deviation in parentheses. **p*<.05; ^*p*<.1. †PP≥.95. Abbreviations. ER = (Bayesian) Evidence Ratio; GAI = Geriatric Anxiety Inventory; GDS = Geriatric Depression Scale; MoCA = Montreal Cognitive Assessment; PP = Posterior probability; WMT = Working Memory Training; tDCS = Transcranial Direct Current Stimulation. The significance of the between-group differences was assessed through generalized modeling, using suitable probability distribution families (and corresponding statistical tests): binomial (gender), negative binomial (Education, MoCA, GAI, GDS), and Conway-Maxwell-Poisson (in all the other cases). Bayesian analysis confirmed all significant and marginal significant frequentist results.

Supplementary Table S2.

*VAS pre and post-tDCS differences between groups for each tDCS session.*

| | | | Frequentist Analysis | | | Bayesian Analysis | | | | |
|---|---|---|---|---|---|---|---|---|---|---|
| **Outcome** | **Day** | **Group comparison** | **Estimate** | **SE** | ***p*-value** | **Estimate** | **EE** | **95%CI** | **ER** | **PP** |
| VAS | 1 | stDCS+WMT –atDCS+WMT | 0.00 | 0.10 | .985 | 0.00 | 0.10 | [-∞ , .17] | 1.05 | .51 |
| | | double-sham – atDCS+WMT | -0.02 | 0.10 | .825 | -0.02 | 0.11 | [-∞ , .16] | 1.39 | .58 |
| | | double-sham – stDCS+WMT | -0.02 | 0.10 | .839 | -0.02 | 0.11 | [-∞ , .16] | 1.34 | .57 |
| VAS | 2 | stDCS+WMT – atDCS+WMT | 0.07 | 0.05 | .137 | 0.07 | 0.05 | [-.01, ∞] | 11.78 | .92 |
| | | double-sham – atDCS+WMT | 0.07 | 0.05 | .111 | 0.07 | 0.05 | [-.01, ∞] | 13.60 | .93 |
| | | double-sham – stDCS+WMT | 0.00 | 0.05 | .917 | 0.00 | 0.05 | [-.07, ∞] | 1.19 | .54 |
| VAS | 3 | stDCS+WMT – atDCS+WMT | -0.02 | 0.05 | .705 | -0.02 | 0.06 | [-∞ , .07] | 1.86 | .65 |
| | | double-sham – atDCS+WMT | -0.06 | 0.06 | .247 | -0.07 | 0.06 | [-∞ , .03] | 6.78 | .87 |
| | | double-sham– stDCS+WMT | -0.04 | 0.06 | .433 | -0.04 | 0.06 | [-∞ , .05] | 3.57 | .78 |
| VAS | 4 | stDCS+WMT – atDCS+WMT | 0.04 | 0.04 | .306 | 0.04 | 0.04 | [-.03, ∞] | 5.16 | .84 |
| | | double-sham – atDCS+WMT | 0.08 | 0.04 | .068^ | 0.07 | 0.04 | [0.00, ∞] | 23.39 | **.96**† |
| | | double-sham– stDCS+WMT | 0.03 | 0.04 | .413 | 0.03 | 0.04 | [-0.04, ∞] | 3.53 | .78 |
| VAS | 5 | stDCS+WMT – atDCS+WMT | -0.03 | 0.03 | .293 | -0.04 | 0.03 | [-∞ , .02] | 5.29 | .84 |
| | | double-sham – atDCS+WMT | -0.04 | 0.03 | .213 | -0.04 | 0.03 | [-∞ , .01] | 8.73 | .90 |
| | | double-sham – stDCS+WMT | -0.01 | 0.03 | .834 | -0.01 | 0.03 | [-∞ , .05] | 1.36 | .58 |

*Note.* ^$p$<.1. †PPS>.95. Significant values in bold. The outcome was analysed as a continuous variable since it was the sum of the individual items. Abbreviations. CI = 95% credible interval. EE = Estimate error. ER = Evidence ratio. PP = Posterior probability. VAS = Visual Analogue Scale.

Supplementary Table S3.

*Hegde's g corrected by baseline for posttest and follow-up (Morris, 2008).*

| | atDCS+WMT *vs* stDCS+WMT | | atDCS+WMT *vs* double-sham | | stDCS+WMT *vs* double-sham | |
|---|---|---|---|---|---|---|
| | Posttest | Follow-up | Posttest | Follow-up | Posttest | Follow-up |
| RAPM set 1 | 0.56 (-0.12; 1.24) | 0.55 (-0.07; 1.17) | 0.53 (-0.16; 1.23) | 0.29 (-0.39; 0.96) | -0.03 (-0.65; 0.58) | -0.26 (-0.92; 0.39) |
| RAPM set 2 | 0.29 (-0.26; 0.83) | 0.17 (-0.34; 0.68) | 0.85 (0.11; 1.59) | 0.64 (-0.07; 1.36) | 0.56 (-0.16; 1.28) | 0.48 (-0.23; 1.19) |
| Bwd Digit Span | 0.41 (-0.17; 0.99) | 0.49 (-0.04; 1.02) | -0.01(-0.55; 0.54) | -0.23 (-0.85; 0.40) | -0.42 (-0.95; 0.10) | -0.72 (-1.29; -0.15) |
| Fwd Digit Span | 0.26 (-0.34; 0.86) | 0.31 (-0.36; 0.98) | 0.14 (-0.38; 0.66) | 0.26 (-0.35; 0.88) | -0.12 (-0.73; 0.48) | -0.05 (-0.67; 0.57) |

*Note.* Confidence Interval between parentheses. Abbreviations. a-tDCS = active tDCS; s-tDCS = sham tDCS; WMT = Working memory training. Bwd = backward. Fwd = Forward. RAPM = Raven's Advanced Progressive Matrices.

Supplementary Table S4.

*Descriptive statistics for the outcome measures by group and time-point (pretest, posttest, follow-up).*

| | | atDCS+WMT | | | | | | stDCS+WMT | | | | | | Double-sham | | | | | |
|---|---|---|---|---|---|---|---|---|---|---|---|---|---|---|---|---|---|---|---|
| | | **Pretest** | | **Posttest** | | **Follow-up** | | **Pretest** | | **Posttest** | | **Follow-up** | | **Pretest** | | **Posttest** | | **Follow-up** | |
| **Task** | **N** | **Mean** | **SD** | **Mean** | **SD** | **Mean** | **SD** | **Mean** | **SD** | **Mean** | **SD** | **Mean** | **SD** | **Mean** | **SD** | **Mean** | **SD** | **Mean** | **SD** |
| RAPM set 1 | 18 | 2.44 | 1.10 | 3.06 | 1.39 | 3.11 | 1.88 | 3.22 | 1.73 | 3.17 | 1.72 | 3.28 | 1.53 | 2.78 | 1.44 | 2.78 | 1.31 | 3.22 | 1.22 |
| RAPM set 2 | 18 | 3.22 | 2.24 | 3.50 | 2.98 | 3.83 | 3.63 | 4.56 | 2.81 | 4.06 | 2.84 | 4.83 | 2.43 | 3.44 | 1.38 | 2.39 | 1.61 | 2.89 | 2.08 |
| RAPM total | 18 | 5.67 | 3.01 | 6.56 | 4.46 | 6.94 | 5.09 | 7.78 | 3.98 | 7.22 | 3.92 | 8.11 | 3.12 | 6.22 | 2.41 | 5.17 | 2.55 | 6.11 | 2.85 |
| Fwd DS | 18 | 7.06 | 1.43 | 7.50 | 1.54 | 7.72 | 1.56 | 7.72 | 1.60 | 7.78 | 1.56 | 7.94 | 1.30 | 8.28 | 2.05 | 8.61 | 1.88 | 8.67 | 2.28 |
| Bwd DS | 18 | 4.83 | 1.72 | 4.94 | 1.86 | 5.00 | 1.88 | 5.83 | 2.12 | 5.06 | 2.18 | 4.94 | 1.89 | 5.61 | 1.50 | 5.72 | 1.74 | 6.11 | 1.37 |

*Note.* Abbreviations. a-tDCS = active tDCS; DS= Digit Span s-tDCS = sham tDCS; WMT = Working memory training. Bwd = backward. Fwd = Forward. RAPM = Raven's Advanced Progressive Matrices.

Supplementary Table S5.

*Pearson correlation coefficients of each transfer measure for pretest-posttest and pretest-follow-up*

| | Pretest *vs* posttest | | | Pretest *vs* follow-up | | |
|---|---|---|---|---|---|---|
| | tDCS + WM | Sham tDCS + WM | Double-sham | tDCS + WM | Sham tDCS + WM | Double-sham |
| Fwd Digit Span | 0.70 | 0.47 | 0.68 | 0.53 | 0.47 | 0.64 |
| Bwd Digit Span | 0.58 | 0.66 | 0.72 | 0.60 | 0.79 | 0.51 |
| RAPM set 1 | 0.41 | 0.58 | 0.54 | 0.60 | 0.58 | 0.43 |
| RAPM set 2 | 0.63 | 0.69 | 0.23 | 0.71 | 0.70 | 0.16 |

Abbreviations. Bwd = backward. Fwd = Forward.

Supplementary Table S6.

*Results of mixed model analysis of near transfer predicting far transfer gains.*

| Near transfer measure | Estimate | Standard Error | *p*-value | Estimate | EE | 95%CI | ER | PP |
|---|---|---|---|---|---|---|---|---|
| DSBA | 0.10 | 0.04 | **.017*** | 0.09 | 0.04 | [.02, ∞[ | 70.43 | **.99**† |
| DSFA | 0.06 | 0.05 | .207 | 0.06 | 0.05 | [-.02, ∞[ | 7.89 | .89 |

*Note.* *$p$<.05. †PS>.95. Significant values in bold. This analysis was performed only for the outcome RAPM_set 1, atDCS+WMT group. Abbreviations. EE = Estimate error. ER = evidence ratio. CI = 95% credible interval. PP = Posterior probability. DS = Digit span. Bwd = backward. Fwd = Forward.

Supplementary Table S7.

*Results of mixed model analysis of individual differences.*

| | | Frequentist analysis | | | Bayesian analysis | | | | |
|---|---|---|---|---|---|---|---|---|---|
| Group | Moment comparison | Estimative | SE | *p*-value | Estimate | EE | CI | ER | PP |
| **AGE (negbinomial)** | | | | | | | | | |
| atDCS+WMT | Posttest – pretest | -0.03 | 0.03 | .270 | -0.03 | 0.04 | ]-∞ , .05] | 3.01 | .75 |
| atDCS+WMT | Follow-up – pretest | -0.05 | 0.03 | **.045*** | -0.06 | 0.04 | ]-∞ , .01] | 9.26 | .90 |
| atDCS+WMT | Follow-up – posttest | -0.02 | 0.02 | .345 | -0.03 | 0.04 | ]-∞ , .05] | 2.69 | .73 |
| stDCS+WMT | Posttest – pretest | -0.04 | 0.02 | .069^ | -0.04 | 0.04 | ]-∞ , .02] | 8.03 | .89 |
| stDCS+WMT | Follow-up – pretest | 0.01 | 0.02 | .618 | 0.01 | 0.03 | [-.04, ∞[ | 1.64 | .62 |
| stDCS+WMT | Follow-up – posttest | 0.05 | 0.02 | **.022*** | 0.06 | 0.04 | [0.00, ∞[ | 15.46 | .94 |
| **EDUCATIONAL LEVEL** (negbinomial) | | | | | | | | | |
| atDCS+WMT | Posttest – pretest | -0.03 | 0.04 | .491 | -0.02 | 0.01 | ]-∞ , .08] | 1.95 | .66 |
| atDCS+WMT | Follow-up – pretest | 0.04 | 0.04 | .306 | 0.04 | 0.06 | [-.06, ∞[ | 2.71 | .73 |
| atDCS+WMT | Follow-up – posttest | 0.06 | 0.03 | .066^ | 0.06 | 0.06 | [-.03, ∞[ | 6.55 | .87 |
| stDCS+WMT | Posttest – pretest | 0.01 | 0.02 | .599 | 0.01 | 0.03 | [-.04, ∞[ | 1.62 | .62 |
| stDCS+WMT | Follow-up – pretest | -0.02 | 0.02 | .389 | -0.02 | 0.03 | ]-∞ , .04] | 2.25 | .69 |
| stDCS+WMT | Follow-up – posttest | -0.02 | 0.02 | .349 | -0.03 | 0.03 | ]-∞ , .03] | 4.05 | .80 |
| **VOCABULARY (negbinomial)** | | | | | | | | | |
| atDCS+WMT | Posttest – pretest | -0.01 | 0.01 | .585 | -0.01 | 0.02 | ]-∞ , .03] | 1.71 | .63 |
| atDCS+WMT | Follow-up – pretest | 0.02 | 0.01 | .149 | 0.02 | 0.02 | [-.01, ∞[ | 4.26 | .81 |
| atDCS+WMT | Follow-up – posttest | 0.02 | 0.01 | **.036*** | 0.02 | 0.02 | [-.01, ∞[ | 8.43 | .89 |
| stDCS+WMT | Posttest – pretest | -0.01 | 0.01 | .321 | -0.01 | 0.02 | ]-∞ , .02] | 2.78 | .74 |
| stDCS+WMT | Follow-up – pretest | -0.02 | 0.01 | **.043*** | -0.02 | 0.02 | ]-∞ , .01] | 7.58 | .88 |
| stDCS+WMT | Follow-up – posttest | -0.01 | 0.01 | .300 | -0.01 | 0.02 | ]-∞ , .02] | 2.63 | .72 |
| **RAVEN (negbinomial)** | | | | | | | | | |
| atDCS+WMT | Posttest – pretest | -0.01 | 0.05 | .837 | -0.01 | 0.08 | ]-∞ , .12] | 1.11 | .53 |
| atDCS+WMT | Follow-up – pretest | -0.01 | 0.05 | .855 | -0.01 | 0.08 | ]-∞ , .12] | 1.19 | .54 |
| atDCS+WMT | Follow-up – posttest | 0.00 | 0.04 | .965 | 0.00 | 0.06 | ]-∞ , .09] | 1.03 | .51 |
| stDCS+WMT | Posttest – pretest | 0.01 | 0.04 | .869 | -0.01 | 0.07 | ]-∞ , .10] | 1.22 | .55 |
| stDCS+WMT | Follow-up – pretest | -0.03 | 0.05 | .472 | -0.04 | 0.07 | ]-∞ , .08] | 2.71 | .73 |
| stDCS+WMT | Follow-up – posttest | -0.04 | 0.05 | .390 | -0.04 | 0.07 | ]-∞ , .08] | 2.29 | .70 |

*Note.* *^$p$<.1, *$p$<.05.* Significant values in bold. CI – 95% credible interval. EE – Estimate error. ER = Evidence ratio. PP – Posterior probability. SE – Standard Error

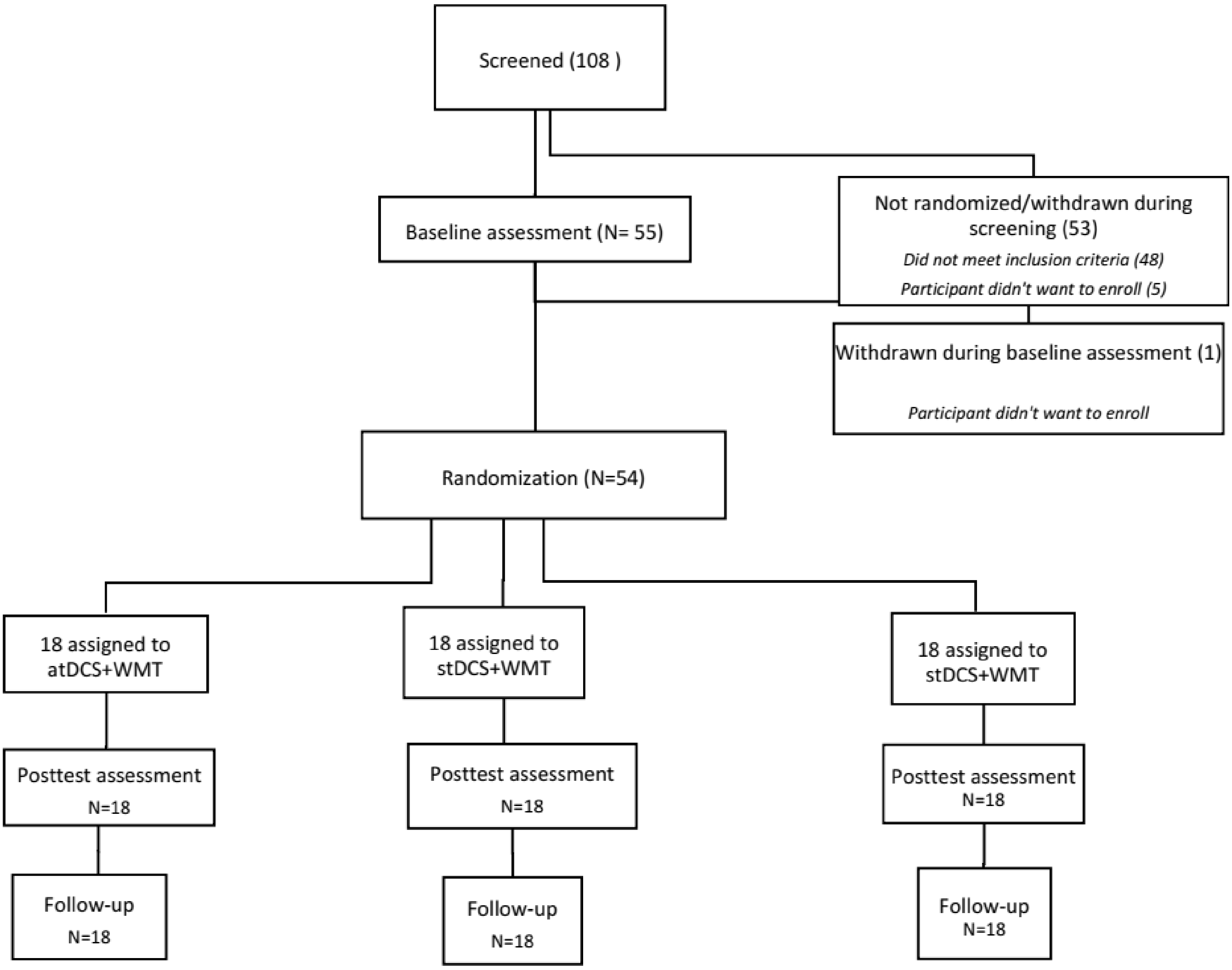


Supplementary Figure S1. Consolidated Standards of Reporting Trials (CONSORT) diagram.

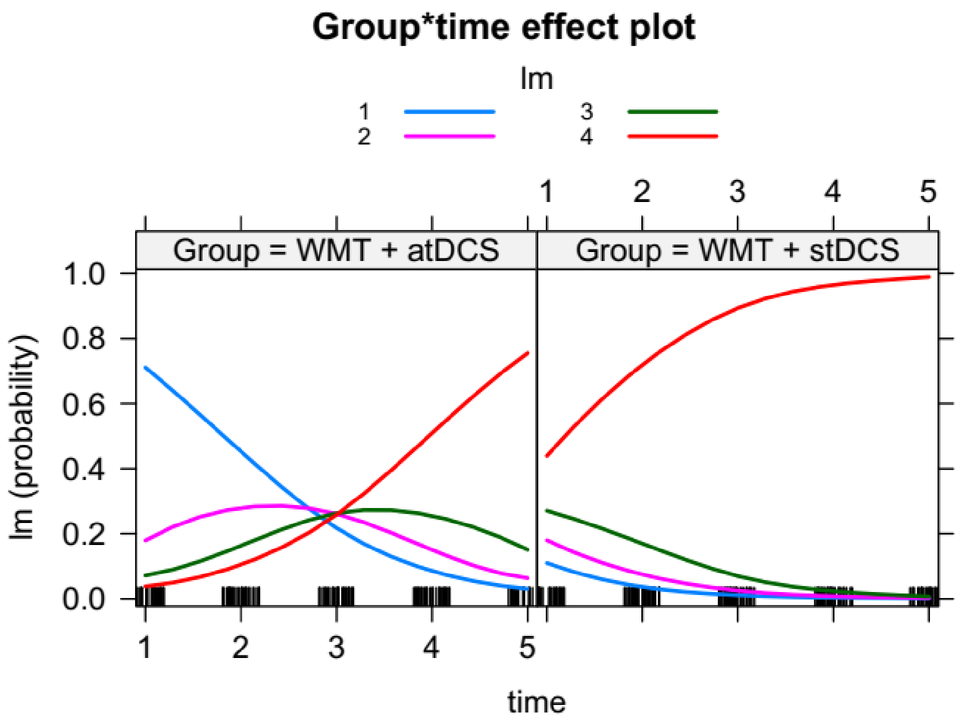


Supplementary Figure S2. Dual *n*-back maximum level (fitted data). This figure shows the probability of participants to achieve a level ≤ 1 (blue), ≤ 2 (pink), ≤ 3 (green) and ≤ 4 (red) in the groups atDCS+ WMT (left) and stDCS+ WMT (right), for each training day. Both groups increased the probability of achieving a level ≤ 4 along the sessions. However, stDCS+WMT started already with a high probability of achieve this level.
